# The Location of Conjunctivochalasis and Its Clinical Correlation with the Severity of Dry Eye Symptoms

**DOI:** 10.3390/medicines5010012

**Published:** 2018-01-22

**Authors:** Georgios Dalianis, Alexandra Trivli, Chryssa Terzidou

**Affiliations:** Department of Ophthalmology, Konstantopouleio-Patission General Hospital, Agias Olgas 3–5, Nea Ionia, 14233 Athens, Greece; g_dalianis@yahoo.gr (G.D.); cterzidou@yahoo.com (C.T.)

**Keywords:** conjunctivochalasis, dry eye, epiphora

## Abstract

**Background:** We aimed to investigate the clinical importance of conjunctivochalasis (CCH) and, further, to implement a new CCH classification system. **Methods:** 60 eyes of patients with whom, upon clinical examination, CCH was diagnosed were investigated for the presence of symptoms and signs characteristic of dry eye. The eyes were grouped based on two stages of severity, Stage 1 (minimal/mild) and Stage 2 (medium/severe), for each nasal, middle, and temporal position, and on the extent of CCH folds in each site. **Results:** In 40 (66.6%) out of 60 eyes, symptoms and signs of CCH were manifest: pain in 25 (41.6%), epiphora in 25 (41.6%), and lacrimal punctum obstruction from conjunctival folds in 22 (36.6%) eyes. Depending on the position of CCH, a greater percentage of symptoms appeared in Stage 2 in the nasal position (78.9%), followed by middle (68.7%) and temporal positions (60%). When TBUT values were compared, statistically significant differences were found proportional to grading (*p* < 0.001) and position (nasal more severe than temporal, *p* < 0.001), and such differences were also found when TBUT values of all eyes were compared with those of symptomatic eyes (*p* = 0.01) and with those of symptom-free eyes (*p* = 0.002). **Conclusions:** CCH is a rather frequent and commonly unrecognized condition that should always be considered in differential diagnoses of dry eye.

## 1. Introduction

Dry eye is common, especially in the elderly. Many disorders with different etiologies and mechanisms may produce dry eye symptoms. Studies in recent years have increased our understanding about the pathology of dry eye and drive therapy on a more orthologic basis [1]. Despite this, symptomatic therapy often cannot alleviate all dry eye symptoms, and refractory cases are met in every day clinical practice with no apparent pathology. Many of these cases are applied in a clinical entity that Elshing in 1908 first described and that Hughes in 1942 named conjunctivochalasis (CCH) [2].

Even though Rieger in 1990 has demonstrated the association between CCH and dry eye [3], and in spite of Tseng’s leading research in this field, the general ophthalmological community is still lacking an understanding of this clinical entity, as the importance of CCH, when present, is ignored in differential diagnoses of dry eye.

Symptoms accompanying CCH depend on the severity and the extent of conjunctival folds that are present, extending from dry eye symptomatology and pain upon ocular movements to pleolacrimal and fluctuating vision. Discontinuation of tear meniscus from conjunctival folds results in precorneal tear film instability, while mechanical obstruction of the lacrimal punctum from excessive conjunctiva increases tear turnover time. Both mechanisms are responsible for symptoms and signs of dry eye or contribute to secondary alterations on the anterior surface [4,5,6].

The objective of this study was to investigate in clinical practice the manifestation of dry eye in patients with CCH.

## 2. Materials and Methods

Thirty consecutive cases (60 eyes) were included in this retrospective study. Participants were recruited when they were admitted for general ophthalmological examination at our service if CCH was apparent on clinical examination. The patients’ ages ranged between 49 and 85 years (mean 77.1 years). Twenty-five of them were women (84.3%), and 5 were men (16.6%). Patients with any kind of anterior surface or lid inflammation as well as obstruction of the lacrimal drainage apparatus were excluded from the protocol. We also excluded patients with very severe CCH where one thick fold was present, overlapping the lid margin.

Patients were examined for the presence of the following: epiphora, pain during eye movements (downward gaze) or blinking, and common symptoms of dry eye (foreign body sensation, burning, or grittiness).

Upon clinical examination, CCH was evaluated and documented by the number of folds and the position on the lid margin (nasal, middle, or temporal). Mechanical lacrimal punctum obstruction due to the nasal folds was evaluated via slit lamp examination, fluorescein staining, fluorescein clearance tests (FCTs), and the presence of epiphora. In patients undergoing pathological FCTs or epiphora, we evaluated the patency of the lacrimal system to rule out any obstruction. Finally, tear break-up time (TBUT) was documented (Table 1).

For the grading of CCH we used a LIPCOF modification, dividing CCH into three stages (Table 2).

**Figure 1:**
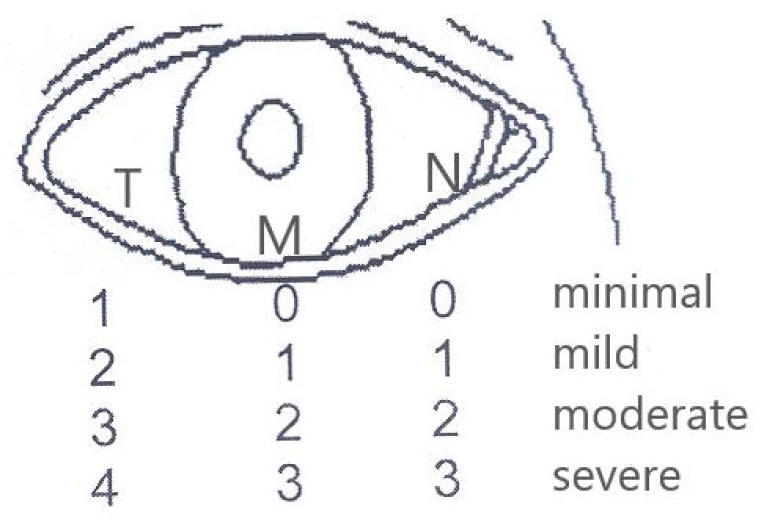
CCH grading.

Stage 1 included minimal and mild chalasis, Stage 2 moderate and severe, and Stage 3 singular large fold resting on the lid margin. Patients with Stage 3 CCH were not included in our study. The severity of CCH was determined by the number of folds present in each of the 3 anatomical regions of the lower lid: nasal (N), temporal (T), and middle (M) (Figure 1). Stage 1 included 1–2 folds temporally and up to 1 folds nasally and centrally, and Stage 2 included 3–4 folds temporally and 2–3 nasally.

The Ethics Committee for Human Research of the hospital approved the study. Ethical approval code: 443/2.11.2017; Date of approval: 29 November 2017. The data were collected by the clinicians who reported the medical records. A written informed consent form was obtained by all participants.

## 3. Results

From the total of 60 eyes, atypical symptomatology was present in 40 eyes (66.6%), and pain was present in 25 eyes (41.6%), while epiphora was present in 25 eyes (41.6%). Occlusion of a punctum from a conjunctival fold was present in 22 eyes (36.6%).

The presence of symptoms (common symptoms and pain, as well as epiphora) was evaluated according to CCH stage in each of the 3 locations (Table 3).

In this way, 38 eyes in nasal, 44 eyes in middle, and 27 eyes in temporal locations were categorized as Stage 1 CCH, while 19 eyes in nasal, 16 eyes in middle, and 30 eyes in temporal locations were evaluated as Stage 2.

According to these results, for Stage 1, the temporal location had a greater percentage of symptoms and epiphora (37% and 38.5%) compared to the nasal location (31.5% and 26.3%, respectively), which had the lowest, while the middle location had percentages between the other two (34% and 31.8%).

In contrast to that, for patients with Stage 2 CCH, the highest percentage of symptoms presented in the nasal location (78.9%, 19 eyes), followed by middle (68.7%, 16 eyes) and temporal (60%, 30 eyes) locations. Regarding epiphora manifestation in Stage 2, the middle location showed a higher percentage (62.5%), followed by nasal (57.8%) and temporal (50%) locations.

In summation, minimal and mild CCH produced atypical symptoms in 27–37% of cases and epiphora in 32–37% of cases, while patients with moderate and severe CCH had atypical symptoms in 60–79% and epiphora in 50–62.5% of cases.

Finally, mean TBUT was compared to the presence of symptoms and the following was observed (Table 4).

Greater TBUT values (mean = 8.23 s) were found in the 20 asymptomatic eyes. The 29 eyes with partial symptomatology had a mean TBUT of 3.91 s, while the lower TBUT values were observed in the 11 eyes with complete symptomatology (mean = 2.8 s). In total, all eyes had low TBUT with a mean of 5.5 s.

Mean TBUT values according to the grading of CCH in each of the 3 locations were compared. In each location, mean TBUT values were getting lower as severity was increasing (Table 5).

More specifically, for the temporal location, mean TBUT was 7.75 s in minimal CCH decreasing to 4.11 s in severe. In the nasal location, mean TBUT ranged from 7.42 to 3.45 s; in the middle location, mean TBUT value at minimal CCH was 6.31 s dropping to 2.66 at severe CCH.

For every location, statistical analysis using Student’s *t*-test (paired) was performed on mean TBUT between Stage 1 and Stage 2 and a statistically significant difference was found between the two groups.

A smaller difference was found in the temporal location (*p* = 0.0014), the largest difference found in the nasal location (*p* = 0.0009), and the middle location was placed in between (*p* = 0.0036)

Eyes with the same stage in each of the 3 locations were considered. Twenty-three eyes were found with Stage 1 and 8 eyes with Stage 2. Concerning the TBUT, the 17 eyes (74%) with Stage 1 that did not have common symptoms had a mean TBUT value of 8.64 s, while the remaining 6 eyes (26%) of Stage 1 that had symptoms had a mean TBUT of 3.66 s. All 8 eyes with Stage 2 had symptoms, and the mean TBUT was 3 s. Statistical significant differences were revealed when mean TBUT values were compared between Stages 1 and 2 (0.032) and between the eyes in Stage 1 with or without common symptoms (*p* = 0.0035) (Table 6).

## 4. Discussion

While CCH has been a recognized pathology since 1908, only during the last several years have there been attempts to investigate the condition and its role in the pathogenesis of dry eye syndrome [7,8,9].

In attempts to explain symptoms manifesting in patients with CCH, we should consider the multiple mechanisms involved, depending on the severity and the position of excessive conjunctiva.

**Figure 2:**
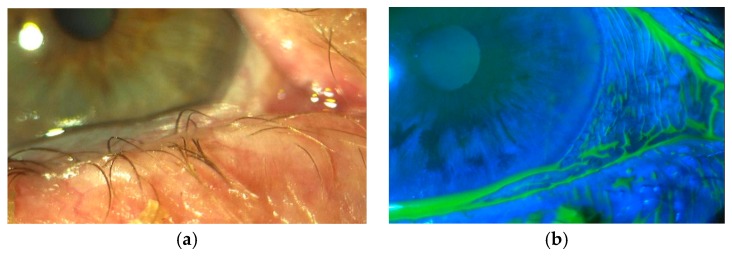
(**a**) Excessive conjunctiva sliding on the cornea. (**b**) After fluorescein staining.

There are two main mechanisms. The first one is regarding tear film instability. Excessive conjunctiva interferes with the normal distribution of the tear film on the cornea either mechanically (sliding on the cornea) or disrupting the integrity of tear meniscus [10] (Figure 2a,b).

Tear film instability is a known cause of dry eye. Severity of symptoms depends on the extent of tear film disorder. Patients with severe tear film instability are more prone to ocular surface disease [1].

The second mechanism is the mechanical occlusion of the lacrimal punctum by excessive conjunctiva, producing intermittent epiphora as well as delayed tear clearance, resulting in the presence of inflammatory components for a prolonged period and the beginning of a vicious circle known as ocular surface disease [1].

Chronic interference of conjunctiva between the bulbar surface and lid margin as well as the absence of proper corneal hydration result in an initial mild inflammatory process that leads to conjunctival epithelium changes and displacement of the Marx line forward. Meibomian glad dysfunction, a possible result of this process, leads to further instability of the tear film, complicating the symptomatology [11].

An already inflamed ocular surface is worsened by the chronic use of medications such as common antibiotics, usually prescribed to relieve the patients’ symptoms. The interaction of these agents, as well as their inactive ingredients and their preservatives, with the ocular surface lead to the continuation of the inflammatory vicious circle [1].

All these findings are supported by our results as well. The percentage of atypical dry eye symptoms and epiphora are increased depending on the severity of CCH. We observed that, when CCH is significant in the middle and nasal region, symptoms are worse, and this can be attributed to the mechanical disruption of tear meniscus, leading to tear film instability as well as the lacrimal punctum occlusion.

Regarding TBUT values, they were decreased in all patients relative to the severity of CCH. Schirmer’s test was not chosen to test dry eye because, apart from being an indicator of tear production with low sensitivity, we considered there would be a high number of false positive results due to delayed tear clearance. TBUT proved to be a valuable indicator for dry eye. We noticed that a lower mean TBUT led to worse atypical symptoms and more severe CCH.

It is not possible for the primary cause of CCH to be determined by this study, but our understanding of the mechanisms by which CCH leads to dry eye can be a helpful tool to relieve symptoms and prevent the appearance of inflammation and the vicious circle to which it leads.

Lid hygiene, the use of anti-inflammatory agents, and artificial tears help with symptomatology. In severe cases, in our experience, the anatomical restoration of the ocular surface with removal of the excessive conjunctiva leads to the disappearance of symptoms, a fact that is also supported in the literature [12,13].

In our study, in order to grade CCH, we modified the Lipcof grading system [14]. It takes into consideration the number of conjunctival folds and their association with tear meniscus height. In this manner, CCH is graded in 4 stages defined as follows: (0) no conjunctival fold; (1) one small fold; (2) more than two folds but not higher than tear meniscus; (3) multiple folds higher than tear meniscus.

We noticed that CCH could be present in all three anatomical regions of the lower lid (temporal, middle, and nasal) without being the same stage in all three regions. Based on our observations we noticed that CCH is initially observed temporally, so Stage 0 is not possible in the temporal region. Furthermore, it was not possible to determine with accuracy the height of tear meniscus compared to the folds due to the increased mobility of the conjunctiva. For these reasons, we divided CCH in 4 stages (minimal, mild, moderate, and severe) for each region (temporal, middle, and nasal) depending on the number of folds and then graded CCH in three stages (Figure 1).

Finally, regarding the incidence of CCH, although our study is not epidemiological, it suggests that CCH is more common in patients of ages higher than 50, while in the literature there are no clear data regarding population and racial or gender distribution. For this reason, we considered the fact that there was a higher percentage of women with CCH in our study (83.4% women and 16.6% men), a random finding.

In conclusion, it is clear from our results that CCH is accompanied by low TBUT values and a wide symptomatology, including the one of dry eye. However, it cannot be determined whether CCH exacerbates an existing dry eye or causes it via the predescribed mechanisms. In either case, it tends to be ignored and should be accounted for in the differential diagnoses of ocular surface disease and dry eye. CCH is a common and possibly overlooked cause of dry eye, which should be treated in order to improve the patient’s quality of life and prevent them from a series of examinations and complicated and prolonged “treatments” that probably intensify the problem.

## Figures and Tables

**Table 1:** Patient data documented on clinical examination.

Symptoms	Findings	Tests
non-typical (itching, burning, foreign body sensation) pain (during blinking or with eye movement) epiphora or pleolacrimal	CCH severity: number and extent of conjunctival folds in each lid position (nasal, middle, temporal). Epiphora (punctum occlusion by overlapping folds).	TBUT FCT

**Table 2:** Staging of conjunctivochalasis (CCH).

Position	Stage 1	Stage 2
	Number of Folds	
T (temporal)	1–2	3–4
N (nasal)	0–1	2–3
M (middle)	0–1	2–3

**Table 3:** Symptoms according to stage per location.

Location	No of Eyes	Epiphora	Atypical Symptoms
Stage 1			
temporal	27	37%	38.5%
middle	44	34%	31.8%
nasal	38	31.5%	26.3%
Stage 2			
nasal	19	57.8%	78.9%
middle	16	62.5%	68.7%
temporal	30	50%	60%
Symptoms: Stage 1: 26.3–38.5% (T > M > N) Stage 2: 50–78.9% (N > M > T)			

T: temporal; M: middle; N: nasal.

**Table 4:** Tear break-up time (TBUT) in eyes with or without symptoms.

Mean TBUT	Eyes/Symptoms
5.50 s	60 eyes
2.80 s	11 complete symptomatology
3.91 s	29 partial symptomatology
8.23 s	20 asymptomatic

**Table 5:** Mean TBUT/CCH severity per location.

CCH Stage	CCH Grading	Mean TBUT		
		Temporal	Middle	Nasal
1	minimal	7.57	6.31	7.42
	mild	5.90	6.08	5.47
2	medium	4.38	4.40	3.87
	severe	4.11	2.66	3.45
*t*-test (Stages 1/2)		Stages 1/2 *p* = 0.0014	Stages 1/2 *p* = 0.0036	Stages 1/2 *p* = 0.0009

**Table 6:** Comparative data of TBUT values in eyes with or without symptoms with the same stage in each of the 3 locations.

Stage/Location		Number of Eyes Symptoms (Yes/No)	TBUT	*t*-Test (Stages 1/2)	*t*-Test (Stages 1/2)
1	23 eyes (T–Μ–N)	17 (74%) NO	8.64 s	*p* = 0.0035	*p* = 0.0032
		6 (26%) YES	3.66 s		
2	8 eyes (T–Μ–N)	8 (100%) YES	3.00 s		
